# Reduction of ADAMTS13 Levels Predicts Mortality in SARS-CoV-2 Patients

**DOI:** 10.1055/s-0040-1716379

**Published:** 2020-08-30

**Authors:** Giovanni L. Tiscia, Giovanni Favuzzi, Antonio De Laurenzo, Filomena Cappucci, Lucia Fischetti, Lazzaro di Mauro, Giuseppe Miscio, Antonio Mirijello, Elena Chinni, Elvira Grandone, on behalf of CSS COVID-19 Group

**Affiliations:** 1 Research Unit of Thrombosis and Hemostasis, Fondazione IRCCS “Casa Sollievo della Sofferenza,” San Giovanni Rotondo, Italy; 2 Transfusion Medicine and Laboratory Department, Fondazione IRCCS “Casa Sollievo della Sofferenza,” San Giovanni Rotondo, Italy; 3 Medical Department, Fondazione IRCCS “Casa Sollievo della Sofferenza,” San Giovanni Rotondo, Italy

In SARS-CoV-2 (severe acute respiratory syndrome coronavirus 2) infected patients, hemostasis shows an uncontrolled activation, with a high D-dimer concentration. 1 2 It has been also shown that the SARS-CoV-2 can promote endotheliitis, 3 and in turn, pulmonary endothelial damage fostering thrombogenesis. 4

Thrombotic microangiopathy (TMA) is defined by microangiopathic hemolytic anemia, thrombocytopenia, and organ failure 5 and can be observed in several disorders. 6 Infectious diseases could be one of these disorders. 7 A disintegrin and metalloproteinase with thrombospondin motifs 13 (ADAMTS13) plays a pathophysiological role in the TMA, including that arousing on sepsis. 8 Moreover, a key role of the ADAMTS13 in the coagulopathy of sepsis is also acknowledged. 9 In the same scenario, ADAMTS13 was shown to be one of the endothelium-related markers showing changes, with a trend toward a reduction. 10 Furthermore, in response to the endothelial activation-induced vascular inflammation, reciprocal changes of the ADAMTS13 and its substrate, the von Willebrand factor (vWF), are observed. 11

In septic patients, reduced synthesis of protein C (PC) and its cofactor protein S (PS) in addition to impaired expression of thrombomodulin and endothelial PC receptor on endothelium determines reduced PC levels with an imbalance of thrombin regulation. 12 Furthermore, antithrombin (AT)— the most important inhibitor of thrombin and activated factor X—is reduced as a consequence of its consumption and impaired synthesis and degradation. 13

We aimed at assessing whether ADAMTS13 levels predict mortality in patients with a diagnosis of SARS-CoV-2 infection. The secondary aim was to investigate a possible relationship between ADAMTS13, D-dimer, vWF, and natural anticoagulants levels to have easy-to-handle markers of clinical outcomes. Seventy-seven patients admitted between March 1 and April 30, 2020 at the “Casa Sollievo della Sofferenza” Research Hospital with a laboratory-confirmed diagnosis (i.e., RT-PCR according to the protocol established by the WHO) 14 of the SARS-CoV-2 infection were investigated. Demographic and clinical information and routine laboratory data at the hospital admission were extracted from hospital electronic medical records. In all patients, ADAMTS13, vWF antigen (vWF:Ag)/functional, and natural anticoagulants (AT, PC, and PS) levels were measured on a blood sample obtained within seven hospitalization days. The ADAMTS13 activity levels were measured using a chromogenic enzyme-linked immunosorbent assay (ELISA) method (TECHNOZYM ADAMTS13 Activity ELISA Kit, Technoclone, Austria). Continuous variables are presented as a median and interquartile range, whereas discrete variables as numbers and percentage. After testing for data normality distribution, Mann–Whitney test was used to analyze differences in continuous variables. Fisher's exact test was also performed to estimate the association between D-dimer positive test (> 500 ng/mL) and ADAMTS13 activity levels below median value. Spearman's *r* was used to explore the reciprocal relationship between ADAMTS13 and D-dimer, vWF, and natural anticoagulants: results are given as *r* - and *p* -values. Multiple linear regression analysis, which controlled for D-dimer concentration, age, and sex was performed to evaluate a predictive model for ADAMTS13 activity levels. Kaplan–Meier analysis was performed to assess whether ADAMTS13 can predict mortality. Statistical significance was set at *p* -values < 0.05. All the analyses were performed using GraphPad Prism version 8.00 for Windows, GraphPad Software (La Jolla, California, United States, www.graphpad.com ). The study was approved by the local ethics committee and was performed according to the Declaration of Helsinki on Ethical Principles for medical research involving human subjects. All patients gave written informed consent.

**Fig. 1:**
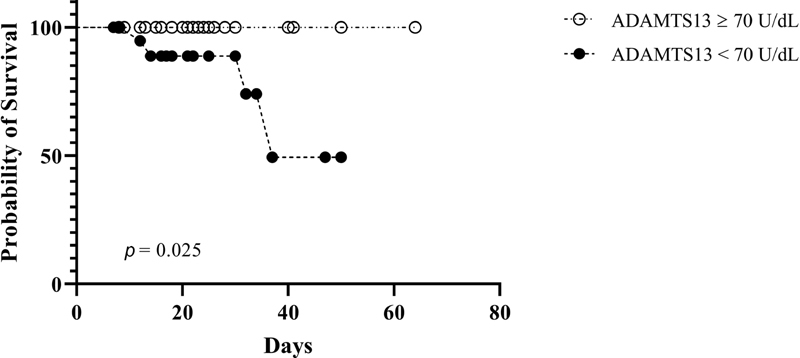
Kaplan–Meier survival analysis according to a disintegrin and metalloproteinase with thrombospondin motifs 13 (ADAMTS13) activity levels: four deaths were observed in the group with values < 70 U/dL.

Descriptive analysis is shown in Table 1 . Most patients (73%) were older than 60 years, with a slightly higher percentage (56 vs. 46%) of women. Almost all the patients (97.5%) showed comorbidities. Fourteen patients (18%) were admitted to the intensive care unit. Median value of ADAMTS13 in the entire sample was 70 U/dL. Kaplan–Meier analysis showed a significantly lower survival in those individuals with ADAMTS13 activity below the median value ( *p* = 0.025) ( Fig. 1 ). Overall, we recorded four deaths (5.2%), all in patients with hypertension, two of them with associated ischemic cardiomyopathy (one diabetic). We cannot exclude that comorbidities could have contributed to mortality in these patients. Seventy-three patients were discharged ( *n* = 51) or are still alive ( *n* = 22) at the end of the study. Since vWF might be directly involved in the pathophysiology of TMA, we performed also the Kaplan–Meier analysis according to vWF ristocetin cofactor (vWF:RCo) levels: no statistically significant difference was found, in terms of mortality prevalence ( *p* = 0.80). Multiple linear regression showed that D-dimer concentration at admission ( *p* = 0.0145) and age ( *p* = 0.0036) were independently associated with ADAMTS13 activity levels. D-dimer concentration above 500 ng/mL at admission was associated with a 2.8-fold higher likelihood of ADAMTS13 activity below the median value (odds ratio: 4.8 [95% confidence interval: 1.3–16.9], *p* = 0.02). Lastly, D-dimer concentration, vWF:Ag, and natural anticoagulants (AT, PC, and PS) levels were inversely (D-dimer and vWF) or directly (AT, PC, PS) associated with ADAMTS13 levels ( Supplementary Table S1 ). ADAMTS13 activity negatively correlated with D-dimer concentration (Spearman's *r* : –0.41, *p* = 0.002 *)* and vWF:Ag (Spearman's *r* : –0.34, *p* = 0.005) levels, whereas a positive correlation was found with AT (Spearman's *r* : 0.45, *p* *<* 0.001), PC (Spearman's *r* : 0.33, *p* = 0.004), and PS (Spearman's *r* : 0.23, *p* = 0.04). The main finding of this study is that patients with ADAMTS13 activity below 70 U/dL have a higher risk of in-hospital death. Present findings are consistent with those observed in patients with sepsis, in whom low ADAMTS13 levels are inversely correlated with vWF 15 and a poor prognosis, as well as with sepsis severity. 16 17 Elevated levels of vWF and reduced ADAMTS13 could correlate with endothelial damage and a progressive worsening of the organ function. In our setting, the ratio of vWF:RCo/vWF:Ag is 0.65. This value does not reflect a predominance of ultra-large vWF multimers, which could represent the culprit of microangiopathic thrombosis. We hypothesize that, at variance with other TMA, the underlying pathogenetic mechanism is the endothelial inflammation and damage resulting in a slight reduction of ADAMTS13 levels. Consistent with this possible pathogenetic mechanism is the lack of thrombocytopenia. Indeed, in TMA other than thrombotic thrombocytopenic purpura, the relationship between reduced ADAMTS13 activity and thrombocytopenia is not clearly demonstrated. 18 As reported in other septic patients, the endothelial damage triggers the coagulation cascade, as suggested by elevated D-dimer and decreased natural anticoagulants plasma levels. 19 20 Notably, we observed that D-dimer, a blood parameter routinely investigated in SARS-CoV-2 patients for the possible clinical impact on mortality in these settings, 21 22 strongly predicts ADAMTS13 levels. Thus, it is conceivable that endothelial dysfunction could be reflected in any of the hemostasis phases and variables, including the natural anticoagulants. Endothelial dysfunction is a key finding and major determinant of poor prognosis in SARS-CoV-2 patients. 23 Indeed, direct or indirect activation by SARS-CoV-2 on endothelium would cause intensive vWF secretion from Weibel–Palade bodies, as expected upon perturbations on these vascular cells. 13 Our data are consistent with previous findings suggesting that SARS-CoV-2 patients present a low-grade disseminated coagulopathy and localized pulmonary microangiopathy, resulting in organ dysfunction. 24 Notwithstanding the retrospective nature and limited size of this study, overall, present findings may speculatively suggest that ADAMTS13 would represent a clinic-diagnostic marker predicting SARS-CoV-2-related prognosis.

**Table 1:** Demographic/clinical information and laboratory data collected in the study population

Variables	Values
Demographic and clinical information	
Age, median (IQR)	61.5 (80–62)
Female/Male	43/34
Coexisting morbidities, *n* (%)	
Any	22 (28.5)
Hypertensive disorder	17 (22)
Chronic renal disease	8 (10.4)
Diabetes	8 (10.3)
Dilated cardiomyopathy	7 (9)
Chronic obstructive chronic disease	7 (9)
Atrial fibrillation	6 (7.8)
None	2 (3)
Deaths, *n* (%)	4 (5.2)
Laboratory data	
ADAMTS13 activity (U/dL), median (IQR)	70 (80–60)
vWF:Ag (%), median (IQR)	231.2 (415.3–205.7)
vWF:RCo (%), median (IQR)	150.0 (334.3–116.9)
ADAMTS13/vWF:RCo, median (IQR)	0.40 (0.50–0.23)
vWF:RCo/vWF:Ag, median (IQR)	0.65 (0.87–0.6)
Factor VIII (%), median (IQR)	95.20 (133.0–69.40)
D-dimer (ng/mL)	882.0 (2250–460.0)
Hemoglobin (g/dL), median (IQR)	11.9 (13.3–9.8)
Platelet count (×10 ^9^ /L), median (IQR)	234 (283–149)
White cell count (×10 ^9^ /L), median (IQR)	6.1 (9.4–4.5)
Lactate dehydrogenase (U/L), median (IQR)	236.0 (314.5–197.0)
Creatinine (mg/dL), median (IQR)	0.9 (1.5–0.6)
C-reactive protein (mg/L), median (IQR)	1.7 (9.3–0.4)
Erythrocyte sedimentation rate (mm/h)	34 (79.5–23.5)
Prothrombin time (International Normalized Ratio), median (IQR)	1.09 (1.18–1.06)
Activated partial thromboplastin time (s), median (IQR)	25.0 (26.5–23.0)

Abbreviations: ADAMTS13, a disintegrin and metalloproteinase with thrombospondin motifs 13; IQR, interquartile range; vWF:Ag, von Willebrand factor antigen; vWF:RCo, von Willebrand factor ristocetin cofactor.
