# Supplementary material for: Reduction of ADAMTS13 Levels Predicts Mortality in SARS-CoV-2 Patients
Source: TH Open. 2020 Aug 30;4(3):e203–6. doi: 10.1055/s-0040-1716379 (PMC7456602; doi:10.1055/s-0040-1716379)
Supplement: Supplementary file 1 — Supplementary Material [file 10-1055-s-0040-1716379-s200057.pdf]

Supplementary Material

Members of the CSS COVID-19 Group

Paolo E. Alboini, Annibale Antonioni, Filippo Aucella, Giovanni Battista Bochicchio, Cristiano Carbonelli, Massimo Carella, Marco Castori, Antonella Centonze, Gianluca Ciliberti, Massimiliano Copetti, Michele Corritore, Salvatore De Cosmo, Leonardo D'Aloiso, Maria M. D'Errico, Angela de Matthaeis, Alfredo Del Gaudio, Annabella Di Giorgio, Vincenzo Giambra, Antonio Greco, Lucia Florio, Andrea Fontana, Vincenzo Inchingolo, Michele Inglese, Maria Labonia, Antonella La Marca, Tiziana Latiano, Maurizio Leone, Evaristo Maiello, Alessandra Mangia, Carmen Marciano, Valentina Massa, Simonetta Massafra, Grazia Orciuli, Nicola Palladino, Rita Perna, Pamela Piscitelli, Matteo Piemontese, Michele A. Prencipe, Pamela Raggi, Maria Grazia Rodriquenz, Raffaele Russo, Daniele Sancarlo, Annalisa Simeone, Vincenzo Trischitta, Michele Zarrelli, Pasquale Vaira, Doriana Vergara, Angelo Vescovi.

Supplementary Table S1 Coagulation markers variation according to ADAMTS13 median

	ADAMTS13 $\geq$ 70 U/dL, $n = 43$	ADAMTS13 $<$ 70 U/dL, $n = 34$	*p*-Value[a]
D-dimer (ng/mL)	635 (1695–419.5)	1797 (3709–883.5)	0.007
vWF antigen (%)	223 (234–197.4)	406 (481.6–206.3)	0.0014
AT (%)	89.8 (98.9–77.3)	75.8 (85.8–59.3)	0.04
PC (%)	110.7 (127.6–95.7)	90.4 (114.5–69.9)	0.003
PS (%)	87.0 (102.7–71.8)	72.1 (86.3–55.1)	0.002

Abbreviations: ADAMTS13, a disintegrin and metalloproteinase with thrombospondin motifs 13; AT, antithrombin; IQR, interquartile range; PC, protein; PS, protein S; vWF, von Willebrand factor.
[a]Mann–Whitney test; data are expressed as median (IQR).
